# Enhanced Emission of Tellurite Glass Doped with Pr^3+^/Ho^3+^ and Their Applications

**DOI:** 10.3390/ma16030925

**Published:** 2023-01-18

**Authors:** Bozena Burtan-Gwizdala, Manuela Reben, Jan Cisowski, Radosław Lisiecki, Bożena Jarząbek, Ali Alshehri, Khalid I. Hussein, El Sayed Yousef

**Affiliations:** 1 Institute of Physics, Cracow University of Technology, ul. Podchorazych 1, 30-084 Cracow, Poland; 2 Faculty of Materials Science and Ceramics, AGH–University of Science and Technology, al. Mickiewicza 30, 30-059 Cracow, Poland; 3 Institute of Low Temperatures and Structure Research, Polish Academy of Sciences, ul. Okolna 2, 50-950 Wroclaw, Poland; 4 Centre of Polymer and Carbon Materials, Polish Academy of Sciences, ul. Sklodowskiej-Curie 34, 41-819 Zabrze, Poland; 5 Physics Department, Faculty of Science, King Khalid University, Abha 61413, Saudi Arabia; 6 Department of Radiological Sciences, College of Applied Medical Sciences, King Khalid University, Abha 61421, Saudi Arabia; 7 Research Center for Advanced Materials Science (RCAMS), King Khalid University, Abha 61413, Saudi Arabia; 8 Department of Medical Physics and Instrumentation, National Cancer Institute, University of Gezira, Wad Medani 2667, Sudan

**Keywords:** tellurite glasses, absorption, emission, rare earth, spectroscopic, Judd–Ofelt, shielding materials

## Abstract

The shielding and spectroscopic properties of Pr^+3^ and Pr^3+^/Ho^3+^-codoped tellurite glass were investigated. The intensity parameters (Ω2 = 3.24-, Ω4 = 1.64-, Ω6 = 1.10 × 10^−20^ cm^2^) as well as the radiative lifetimes of ^3^F_4_ + ^5^S_2_ and ^5^I_6_ excited states of Ho^3+^ ions were equal to 301 μs and 3.0 μs, respectively. The former value appears to be much higher than that obtained from the lifetime measurement, indicating the presence of various energy transfer processes. The NIR spectrum of Pr^3+^/Ho^3+^-co-doped tellurite glass is dominated by strong Ho^3+^: ^5^I_6_ emission at around 1200 nm, being the result of the energy transfer from Pr^3+^ to Ho^3+^ ions. The shielding effectiveness of the prepared glasses showed good performance against high-energy photons. These findings suggest that the prepared glasses could be used in laser technology such as photodynamic therapy (PDT) treatment procedures and as shielding for radiation protection.

## 1. Introduction

Tellurite glasses with rare earth (RE) doping are promising materials for photonic applications such as solid-state lasers and lighting, optical amplifiers, and medical and sensing technologies. The applications of tellurite glasses are connected with their high transmittance from the visible to mid-infrared ranges (0.4–6 μm), high refractive index (*n* > 2), and low maximal phonon energy (~750 cm^−1^) [1,2]. One of the most frequently used dopants for tellurite glasses is the Ho^3+^ ion, which exhibits both visible and infrared emissions due to its unique energy level structure [3]. In particular, these materials are expected to show an emission in the IR region at 1.2 μm, corresponding to the ^5^I_6_ → ^5^I_8_ transition. In addition, the emissions are also observed at 1.38 and 1.46 μm, for the ^5^F_4_+^5^S_2_→^5^I_6_ and ^5^F_5_→^5^I_6_ transitions, respectively. Emission from levels ^5^I_6_ → ^5^I_8_ and ^5^F_4_+^5^S_2_ → ^5^I_5_ of Ho^3+^ ions may be promising for obtaining a signal band gain of 1.2–1.4 μm [4,5,6].

Ho^3+^ions can induce interesting emission and luminescence decays. Therefore, glasses doped with Ho^3+^ions exhibit visible green and red emissions at exited level (^5^F_4_, ^5^S_2_)→^5^I_8_, and ^5^F_5_→^5^I_8_, respectively [6,7,8,9,10]. The relationship between green and red emission intensities strongly depends on the base matrix and the concentration of Ho^3+^ ions. Host materials containing Pr^3+^ ions have a broader luminescent range than other trivalent rare-earth ions [10,11]. Glasses that are co-doped with holmium and praseodymium have been studied as sources of applications of lasers in the mid-infrared and white light radiation. Double doping with Pr^3+^ and Ho^3+^ ions is mainly used to increase mid-infrared emissions. As for NIR emission, which is usually weak for Ho^3+^-only doped materials, a significant increase in PL intensity can be achieved by co-doping with Pr^3+^ ions, as signaled in Ref. [12]

In addition to the enhanced luminescence properties, the tellurite glasses have been widely investigated for different medical radiation applications such as shielding material and photodynamic therapy (PDT) treatment procedures [13,14,15,16,17,18,19,20,21,22]. For use in photodynamic therapy (PDT) treatment procedures and medical radiation technologies, laser glasses doped with rare earth elements such (Pr^3+^, Sm^3+^, Ho^3+^, and Tm^3+^) have been developed [20,21,22]. Light sources for PDT include incandescent lamps and xenon lamps with appropriate filters. However, their progress and use are limited by the relatively low light intensity of these sources, making lasers, which can be precisely directed and produce strong light, the preferred illumination method of choice. For application as a safety replacement device in medical workplaces such as PDT, X-ray, and atomic projects in the area of innovations, V-ray equipment, gamma camera rooms, and computed tomography (CT) examination workplaces, researchers analyzed the lasing and shielding qualities of such glasses [21,22]. According to J. Yang et al. [21], the radiant flux and quantum yield for the red fluorescence of Pr^3+^ is calculated to be 219W and 11.80%, respectively, under the commercial blue LED illumination. The excitation band of the majority of photosensitizers (PS) matches the wavelength range of 85.24% of the fluorescence photons in the visible region, showing significant potential for photodynamic therapy (PDT) treatment and clinical studies [21].

The aim of this study is to investigate spectroscopic analyses of novel, multicomponent tellurite glasses co-doped with Pr^3+^/Ho^3+^, to be employed as a laser material in radiology rooms for photodynamic therapy surgery (PDT). Moreover, the shielding effectiveness of the prepared glasses has been investigated due to the advantages of tellurite glasses doped rare earth ions as shielding material.

## 2. Experimental Work

The core six tellurite glass samples were prepared by melting 25 g batches of highly pure (99.99%) chemicals in gold crucibles at 850 °C in an ambient atmosphere. The resulting material has the molar composition 78TeO_2_- 10Nb_2_O_5_- 5PbO- 1PbF_2_- 5Li_2_O-1La_2_O_3_. To prevent vaporization losses, a platinum plate was placed on top of the crucibles. While melting, the melts were periodically agitated to prevent inhomogeneity. The melts were then placed onto plates that had been warmed to 400 °C, generating layers that were a few mm thick, and these layers were then annealed between 320 and 340 °C. The concentrations of lanthanide ions (N_Ln_) of Pr_2_O_3_ and Ho_2_O_3_ incorporated into the host matrix have been calculated by the equation reported in Ref [23]. The density of the prepared glasses was determined using Archimedes’ law [23]. The glass samples were sliced and polished to a size of around 5’5’2 mm^3^ for spectroscopic measurements. The M-2000 Woollam ellipsometer was used to collect the ellipsometric data, and the Perkin Elmer Lambda 900 spectrophotometer was used to capture the transmittance and reflectance spectra. The luminescence decay curves were obtained after a short pulse stimulation delivered by an optical parametric oscillator powered by a third harmonic of a Nd:YAG laser [24]. The photoluminescence spectra were measured using an Optron Dong Woo fluorometer system. Table 1 shows the sample code, composition, molar mass, density, and concentrations of lanthanide ions. The shielding parameters of the prepared samples such mass and linear attenuation coefficients (MAC and LAC), half-value layer (HVL), and mean free path (MFP), were investigated using MIKE software [25].

### Shielding Properties 

The possibility of photons interacting with a barrier is characterized as the mass attenuation coefficient (MAC) of a shielding material, and it is expressed as follows for a combination of elements or any chemical molecule [26].

(1) $$\mathrm{MAC}={\;\mu }_{m}=\overset{n}{\underset{i=1}{\sum }}w_{i}{\left(\frac{\mu }{\rho }\right)}_{i}$$

where *w_i_* represents the fractional weight of individual components in each compound, and ρ  indicate the density of material, µ/ρ is mass attenuation of the individual components. The LAC can then be estimated by using the following relationship:

(2) LAC=MAC ×ρ 

The average distance a photon can go through the barrier without interacting is known as the mean free path (MFP), and it is determined by the reciprocal of the linear attenuation [27]:

(3) $$\mathrm{MFP}=\frac{1}{\mathrm{LAC}\left(\mu \right)}$$

The thickness of the interaction target at which the attenuated intensities account for 50% of the narrow photon beam intensity is defined by the half-value layer (HVL). The necessary thickness of shielding material is inversely proportional to the HVL value. The HVL can be calculated using the following equation [27]:

(4) $$\mathrm{HVL}=\frac{\ln2}{\mathrm{LAC}\left(\mu \right)}=\frac{0.693}{\mathrm{LAC}\left(\mu \right)}$$

## 3. Results and Discussion 

### 3.1. Absorption Spectra and Judd–Ofelt Analysis

**Figure 1:**
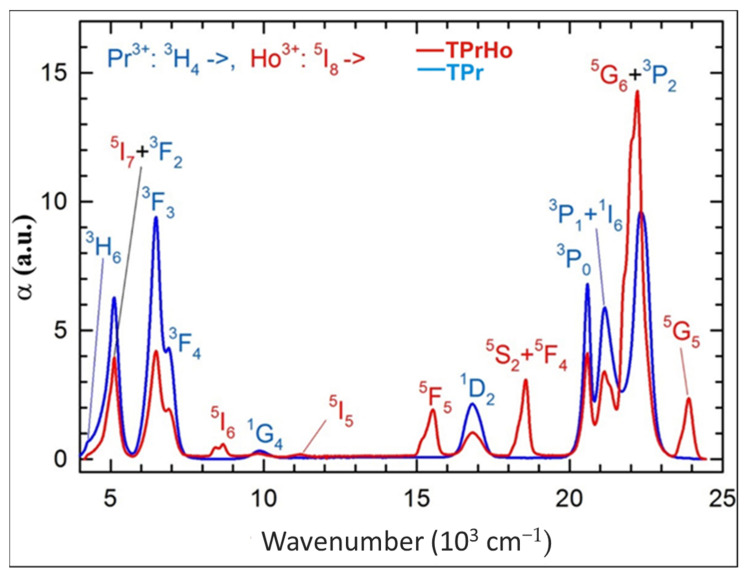
Net absorption spectra of samples TPr and TPrHo.

Figure 1 shows the net absorption bands of Pr^3+^ and Ho^3+^ ions, obtained by subtraction of the monotonic background absorption of the glass matrix (shown for sample T0 in [28]) from the total absorption determined for samples TPr and TPrHo.

As seen in Figure 1, the spectra of samples TPr and TPrHo show the existence of several single absorption bands attributed to transitions from the ground states of both ions, Pr^3+:3^H_4_ and Ho^3+:5^I_8_, to subsequent excited states, as indicated elsewhere [29].

In addition, the spectra of sample TPrHo show overlapping absorption bands associated with transitions to excited levels of Ho^3+:5^I_7_ and Pr^3+:3^F_2_ in the NIR region as well as transitions to levels of Ho^3+:5^G_6_ and Pr^3+:3^P_2_ in the visible region.

**Figure 2:**
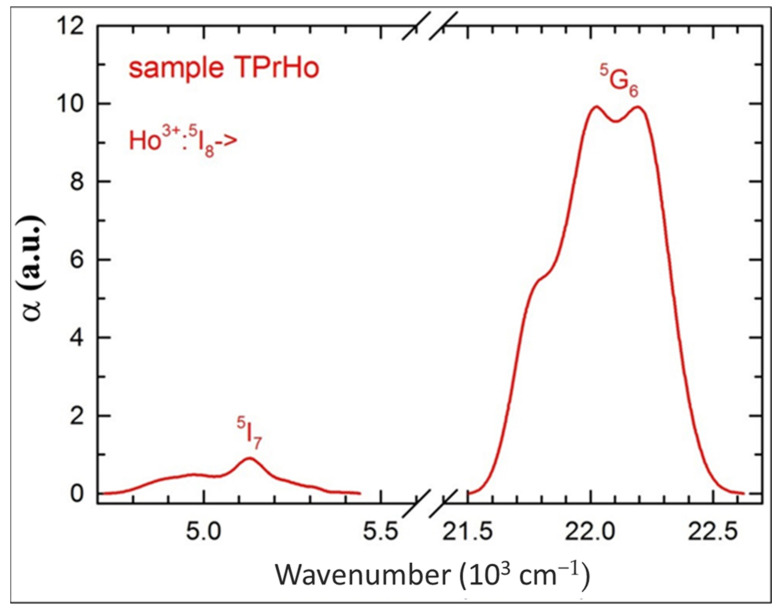
Extracted absorption bands of Ho^3+^ ions for sample TPrHo.

One can see in Figure 1 five Ho^3+^ absorption bands, that do not overlap with Pr^3+^ bands allowing one to perform the standard J-O analysis for the former ion. However, it appears that such an analysis results in a negative, unphysical value of the Ω_2_ J-O intensity parameter, due to the neglect of the strong Ho^3+^:^5^G_6_ absorption band. In order to include this band, we have subtracted from the sample TPrHo absorption a contribution from the Pr^3+^ absorption, which is equal to half of that measured for sample TPr, considering the difference in Pr^3+^ ion concentrations between the two samples as shown in Table 1. For completeness’ sake, we have also extracted the Ho^3+:5^I_7_ absorption band, and both bands are shown in Figure 2.

It appears that the extracted absorption bands from Figure 2 are very similar to those determined for a single Ho^3+^-doped tellurite glass [30], justifying the extraction procedure. 

Finally, we have applied the standard Judd–Ofelt theory to six electric dipole transitions specified in Table 2, calculating their oscillator strengths by numerical integration of the corresponding absorption bands; in order to minimize the root-mean-square deviation, the transition ^5^I_8_→^5^I_7_ has been omitted in the analysis due to a significant, magnetic dipole contribution to the oscillator strength and uncertainty connected with the extraction procedure [31]. The oscillator strength values of the prepared glass were calculated using Equation reported in Ref [23].

The refractive index dispersion of samples T0 and TPr, obtained from ellipsometric data, is presented in our previous work [28], as being very well described by the Sellmeier model of the form n(λ) = [A + Bλ^2^/(λ^2^ − C^2^) − Dλ^2^]^1/2^, where A, B, C, and D are the fitting parameters. Adopting the same procedure for sample TPrHo, the values of the fitting parameters have been found as *A* = 2.729, *B* = 1.764, *C* = 244.16 nm, and *D* = 2.52 × 10^−9^ nm^−2^. As for the reduced matrix elements for the electric dipole transitions, we have used the data from Ref. [32]. Table 2 shows the calculated Judd–Ofelt parameter values along with the root-mean square deviation (*δ_rms_*).

The obtained values of the Ω*_i_* (*I* = 2,4,6) parameters follow the same pattern as those observed for different Ho^3+^ -doped tellurite glasses and follow the same sequence, namely Ω_2_ > Ω_4_ > Ω_6_ [29]. On the other hand, the sequence is different with Tellurite glasses doped with Pr^+3^ [24,28].

The J-O parameters have been used to estimate the transition probability *W_r_* of ^5^I_6_ (323 s^−1^), ^5^S_2_ (4549 s^−1^) and ^5^F_4_ (2060 s^−1^) excited states of Ho^3+^ ions and subsequently the total radiative lifetimes *τ_rad_* = 1/*W_r_*. The levels ^5^S_2_ (lower) and ^5^F_4_ (higher) are very close to each other, leading to one absorption band, as shown in Figure 1. The occupation of these levels is governed by the Boltzmann factor, exp(-Δ*E*/*k_B_T*), where Δ*E* is the energy separation between the levels, *k_B_* is the Boltzmann constant, and *T* is temperature. To calculate Δ*E,* we have fitted two Gaussians to the (^5^S_2_ + ^5^F_4_) absorption band, which yields Δ*E* = 128 cm^−1^. The common transition probability for these levels is given by [33]

(5) $$W_{r}\left(\;J^{\prime }+J\prime \prime \right)=\frac{\left(2J^{\prime }+1\right)W_{r}\left(\;J^{\prime }\right)+\left(2\;J\prime \prime +1\right)W_{r}\left(\;J\prime \prime \right)\exp\left(-\Delta E/k_{B}T\right)}{\left(2J^{\prime }+1\right)+\left(2\;J\prime \prime +1\right)\exp\left(-\Delta E/k_{B}T\right)},$$

where J′ = 2 and J″ = 4 are the total momenta of ^5^S_2_ and ^5^F_4_ excited levels, respectively.

### 3.2. Emission Spectra

In addition, the PL signal of both samples (in arbitrary units) has been divided by *N_Pr_*; to illustrate the impact of Pr^3+^ ion concentration (*N_Pr_*) on the emission spectra as shown in Table 1. Emission spectra were obtained under identical conditions, permitting comparison of their relative intensities.

**Figure 3:**
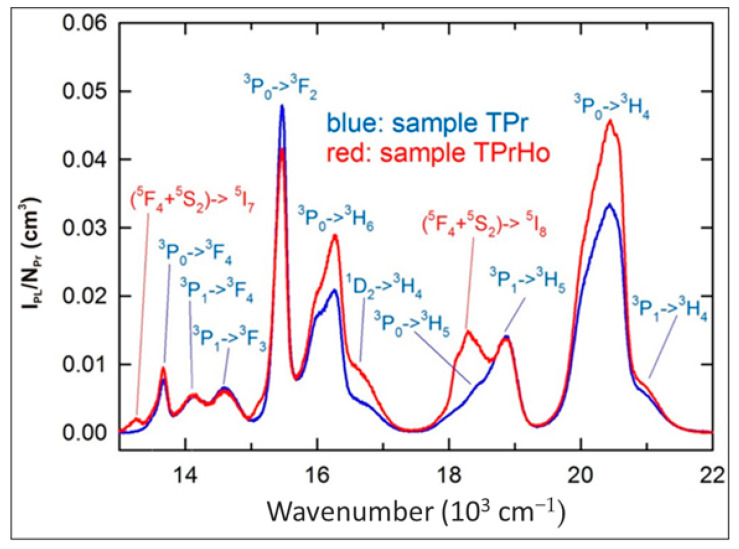
Visible emission spectra of sample TPr and TPrHo (λ_exc_ = 445 nm).

A significant number of excited states of Pr^3+^ and Ho^3+^ ions and their relatively high concentrations in the investigated samples create many possible radiative and non-radiative energy transfer (ET) processes between the Pr-Pr, Ho-Ho, and Pr-Ho pairs as discussed below. Figure 3 shows the visible PL spectrum for samples TPr and TPrHo under 445 nm (22,472 cm^−1^) excitation, corresponding to Pr^3+^: ^3^H_4_→^3^P_2_ and Ho^3+^: ^5^I_8_→^5^G_6_ absorption bands, as shown in Figure 1.

**Figure 4:**
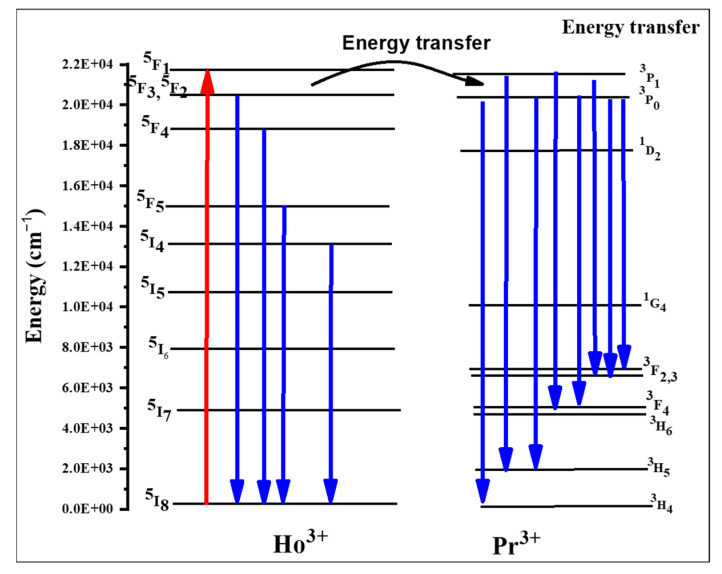
The energy level diagram of Ho^+3^/Pr^+3^ ion.

When comparing the emission spectra from Figure 3, it is clear that the spectrum of sample TPrHo consists of many Pr^3+^ emission bands that are completed with two Ho^3+^ bands, namely with a relatively strong (^5^F_4_+^5^S_2_)→^5^I_8_ emission at 18,290 cm^−1^ and a weak (^5^F_4_+^5^S_2_)→^5^I_7_ emission at 13,250 cm^−1^. With the exception of the ^3^P_0_→^3^H_6_ and ^3^P_0_→^3^H_4_ bands, many Pr^3+^ emission bands are generally proportional to NPr, indicating that the concentration has been quenched by cross-relaxation and energy migration processes [30,34]. Similar cross-relaxation pathways result in numerous transitions between the energy levels of this ion in glasses doped exclusively with Ho^3+^ ions [30,35,36,37,38]. The energy level diagram of Ho^+3^/Pr^+3^ ion codoped in the present host matrix were shown in Figure 4.

**Figure 5:**
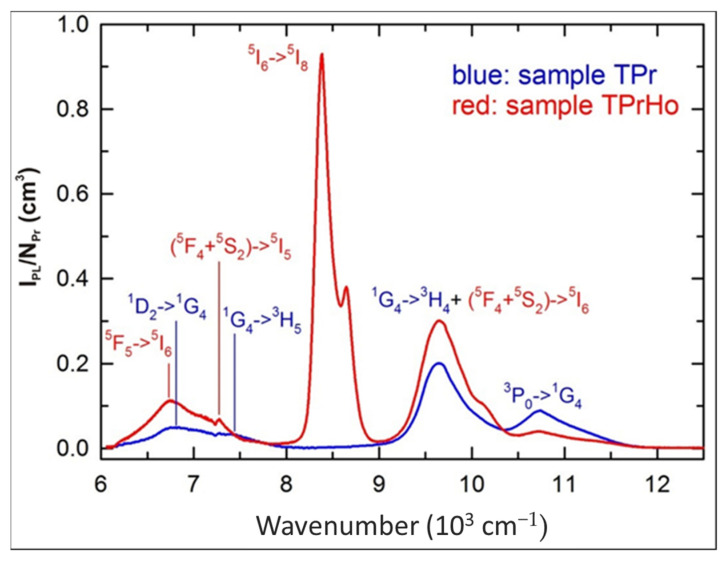
NIR emission spectra of samples TPr and TPrHo (λ_exc_ = 445 nm).

Figure 5 displays the NIR emission spectra of the samples TPr and TPrHo, normalized as scale from the visible spectra in Figure 3.

For the sample TPrHo, the weak overlapping emission bands of prepared glasses doped with Pr^3+^ and Pr^3+^/Ho^3+^ ions in the 6000–8000 cm^−1^ region were attributed. Furthermore, beyond 9000 cm^−1^, there is another overlapping emission band of present glasses that appeared at 9648 cm^−1^. The sample TPrHo spectra is characterized by strong emission at 8386 cm^−1^ attributed to Ho^3+^: ^5^I_6_ transition. When comparing the emission of sample TPrHo to the transition Pr^3+^:^3^P_0_→^1^G_4_ emission from sample TPr at 10,735 cm^−1^, the energy transfer (ET) from Ho^3+^ to Pr^3+^ ions is observed. After 445 nm, the photons excite the Pr^3+^:^3^P_2_ level, and there is a non-radiative transition to the ^3^P_0_ level, followed by cross-relaxation and the ^1^G_4_ level as an intermediate state. This mechanism populates the Ho^3+^:^5^I_6_ level by the pathways Pr^3+^(^3^P_0_→^1^G_4_)→Ho^3+^(^5^I_8_→^5^I_6_) and Pr^3+^(^1^G_4_→^3^H_4_)→ Ho^3+^(^5^I_8_→^5^I_6_). The Ho^3+^:^5^I_6_→^5^I_8_ emission, which is preceded by the non-radiative and radiative transitions ^5^G_6_→(^5^F_4_+^5^S_2_) and (^5^F_4_+^5^S_2_)→^5^I_6_, is amplified by this down-conversion mechanism [35].

### 3.3. Lifetime

Table 3 includes the lifetimes of the ^3^P_0_ excited state of the Pr^3+^ ions as well as the predicted lifetime values and experimental data in order to compute the ET efficiency from these ions to Ho^3+^ ions. The ^3^P_0_ Lifetimes of TPr are taken from our previous work [28]. The (^3^F_4_+^5^S_2_) lifetime of TPrHo was measured and calculated as shown in Table 3. The ^5^I_6_ lifetime is only calculated in the present study.

**Figure 6:**
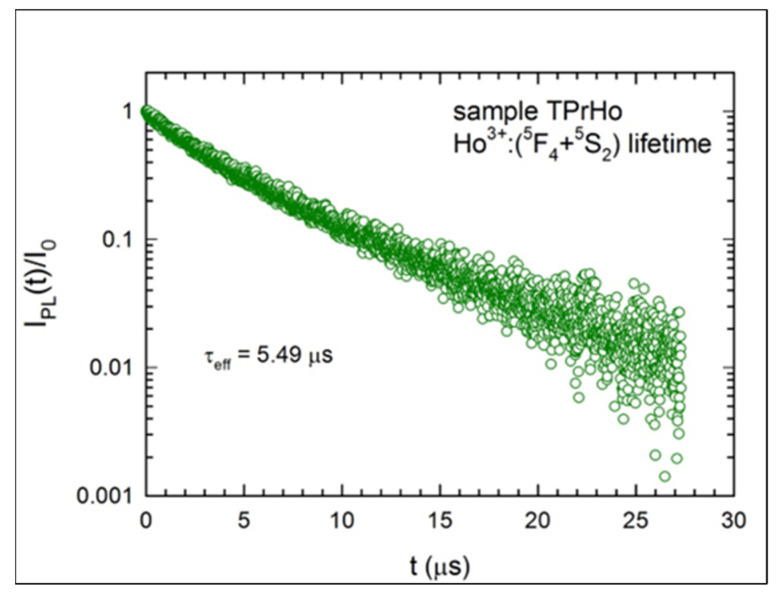
Luminescence decay of the Ho^3+^:(^5^F_4_+^5^S_2_) emission for sample TPrHo.

**Figure 7:**
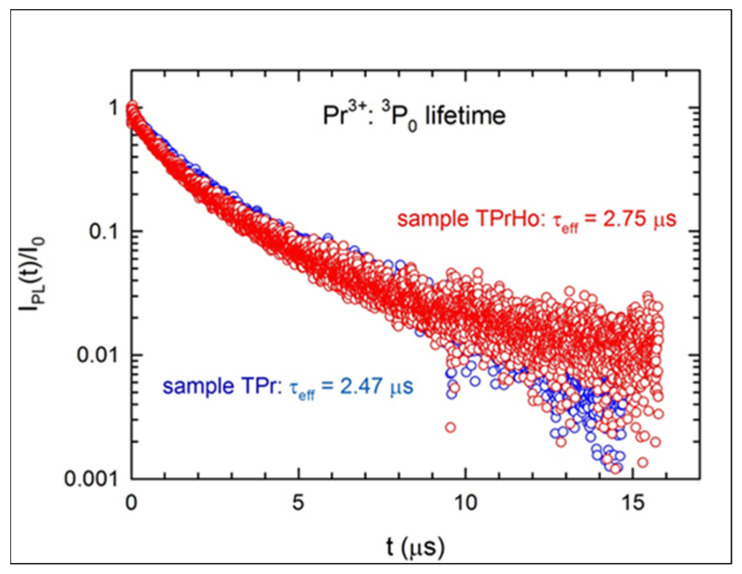
Luminescence lifetimes of the Pr^3+^:^3^P_0_ emission for samples TPr and TPrHo.

Figure 6 and Figure 7, respectively, show the excited levels of Ho^3+^:(^5^F_4_ + ^5^S_2_) and Pr^3+^:^3^P_0_’s PL decays. Since the decay data are non-exponential, we have established that the experimental lifespan is equal to the effective lifetime, which is defined as follows:

(6) $$\tau_{\mathrm{eff}}=\frac{\int t\;\times I\left(t\right)\mathrm{dt}}{\int \mathrm{tI}\left(t\right)\mathrm{dt}}.$$

There are numerous potential energy transfer mechanisms between the ions when Pr^3+^ and Ho^3+^ ions are present in sample TPrHo at relatively high and comparable concentrations. As a result, the measured lifetime of Ho^3+^:(^5^F_4_ + ^5^S_2_) emission is significantly shorter (5.49 μs) than that predicted from the J-O analysis (301μs; Table 3).

As was mentioned before while describing the sample’s emission spectra, sample TPr has a high concentration of Pr^3+^ ions, which results in an effective cross-relaxation (CR). When compared to the J-O radiative lifetime (9.43 μs [28]), the Pr^3+^:^3^P_0_ effective lifetime is significantly reduced by the CR mechanism, going down to *τ_eff_* = 2.47 μs. The concentration of Pr^3+^ ions in sample TPrHo is two times lower than in sample TPr, so one would expect that *τ_eff_* would be significantly higher and equal to 3.92 μs [28]. However, when the former sample is codoped with Ho^3+^ ions, an additional energy transfer (ET) occurs between the two ions, which reduces the Pr^3+^:^3^P_0_ lifetime to 2.75 μs.

The ET efficiency (η) from Pr^3+^ to Ho^3+^ ions can be estimated using the following equation, calculated as [29]

(7) $$\eta =1-\frac{\tau_{Pr-\mathrm{Ho}}}{\tau_{Pr}}\;$$

where *τ_Pr-Ho_* and *τ_Pr_* are the effective lifetimes with and without Ho^3+^ co-doping, respectively, for the same concentration of Pr^3+^ ions. Using the value *τ_Pr_* = 3.92 μs, corresponding to *N_Pr_* =3.16×10^20^ cm^−3^, we have obtained the ET efficiency η = 29% that can be compared with the values 19 and 32.3% found for tellurite glasses doped with 1 mol% of Pr^3+^ ions, and codoped with 0.5 and 1 mol% of Ho^3+^ ions, respectively [28]. The observed increase of ET efficiency with the increasing N_Ho_/N_Pr_ ratio is in accordance with the theoretical modeling of quantum cutting via a two-step ET [39].

### 3.4. ^5^I_8_→^5^I_6_ Transition

The absorption cross-section (ACS) and emission cross-section (ECS) of the ^5^I_8_↔ ^5^I_6_ transition are the key characteristics for use in optoelectronics, thus we will focus on them.

The absorption cross-section (ACS; *σ_abs_*) of the ^5^I_8_→^5^I_6_ transition was determined in the range of 1100–1250 nm by dividing the absorption coefficient by the Ho^3+^ ion concentration. This makes it possible to use the McCumber formula to determine the emission cross-section (ECS; *τ_em_*) of the ^5^I_6_→^5^I_8_ transition [40].

(8) $$\sigma_{\mathrm{em}}^{\mathrm{MC}}\left(\lambda \right)=\exp\left(\frac{E_{\mathrm{mt}}}{k_{B}T}\right)\sigma_{\mathrm{abs}}\left(\lambda \right)\exp\left(-\frac{\mathrm{hc}}{{\lambda k}_{B}T}\right),$$

where h is the Planck constant., E_mt_ is the mean transition energy between the ^5^I_8_ and ^5^I_6_ levels, which can be calculated by averaging the barycenter energies of the absorption and emission spectra. This results in E_mt_ = 8519 cm^−1^. 

As for the measured ^5^I_6_→^5^I_8_ emission, it is given in arbitrary units in Figure 5. To scale this emission, we have used the equation for ECS following the Füchtbauer–Ladenburg (F-L) approach and given by (see Ref [9])

(9) $$\sigma_{\mathrm{em}}^{F-L}\left(\lambda \right)=\frac{\lambda^{5}W_{r}\beta }{8{\pi \mathrm{cn}}^{2}}\cdot \frac{I\left(\lambda \right)}{\int \lambda I\left(\lambda \right)d\lambda },$$

with the branching ratio *β* = 0.91 and *W_r_* = 323 s^−1^ that were calculated for ^5^I_6_ → ^5^I_8_ emission from the J-O analysis.

**Figure 8:**
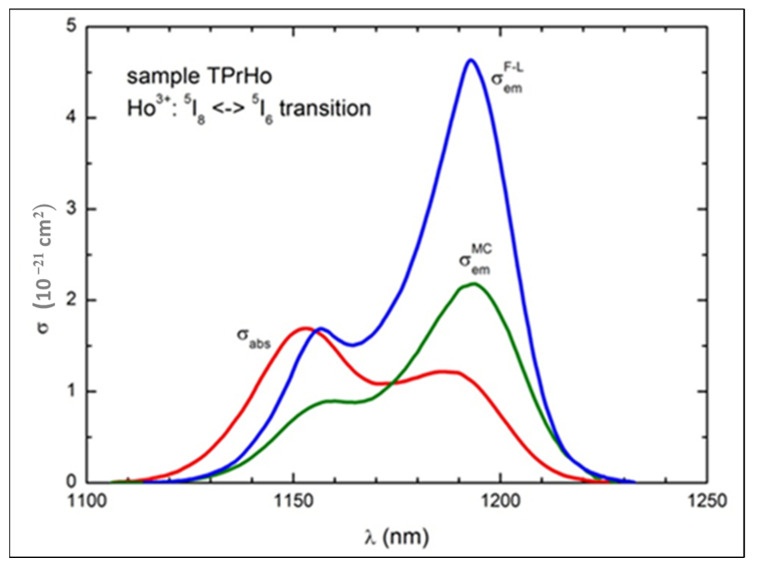
The experimental absorption cross-section (*σ_abs_*) and the emission cross-sections $\sigma_{em}^{MC}$ $\mathrm{and}\;\sigma_{em}^{F-L}$ calculated for Ho^3+^: ^5^I_6_→^5^I_8_ emission using Equations (8) and (9), respectively.

The ACS and ECS spectrums were calculated using Equations (8) and (9). The calculated spectrum is shown in Figure 8.

As shown in Figure 8, there is a relatively small shift (107 cm^−1^) between the peak value of ACS at 1167 nm (10,246 cm^−1^) and that of ECS at 1181 nm (10,163 cm^−1^). The McCumber and Füchtbauer–Ladenburg (F-L) curves are quite similar in shape but very different in magnitude; the peak value of $\sigma_{em}^{F-L}\;$(4.63 × 10^−21^ cm^2^) is about two times greater than that of $\sigma_{em}^{MC}$ (2.18 × 10^−21^ cm^2^), suggesting effective ET from Pr^3+^ to Ho^3+^ ions, since the MC curve reflects emission of Ho^3+^ ions in the absence of Pr^3+^ ions, while the F-L curve represents the emission enhanced by codoping with the latter ions [39,40]. 

### 3.5. Shielding Properties

**Figure 9:**
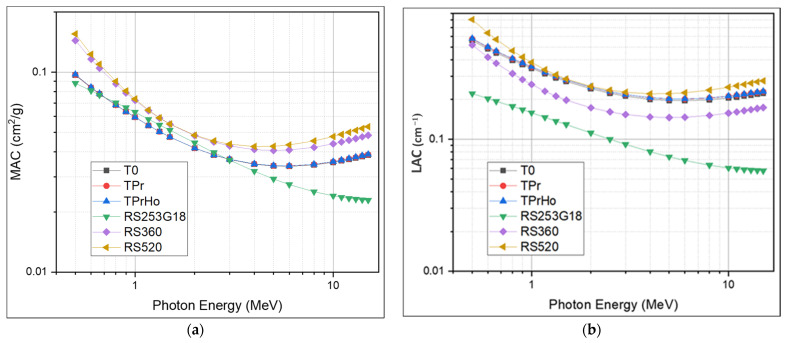
(**a**) The mass attenuation coefficient of prepared glasses; (**b**) The linear attenuation coefficient of the prepared glasses.

Over a broad energy range, ranging from 0.5 to 15 MeV, the shielding effectiveness of the produced glasses was examined. Using MIKE software, the radiation parameters of the investigated glasses were estimated. The mass attenuation coefficient and linear attenuation coefficients were investigated in the intermediate and high photon energy ranges, ranging between 500 keV and 15 MeV. The results of the mass attenuation coefficient and linear attenuation coefficients of the prepared glasses were compared to those of commercially available standard materials coded RS-253 G18, RS-520, and RS-360 [41], as shown in Figure 9a,b. As illustrated in Figure 9, the values of MAC and LAC decrease slowly as the photon energy increases. This trend is in fact due to the dominance of Compton scattering, which is directly proportional to the atomic number and inversely proportional to the photon energy [42]. As the photon energy increases above 5 MeV, a slight increase in the MAC and LAC is observed. This trend is mainly due to the contribution of the pair production process, which is directly proportional to the square of the atomic number and directly proportional to the photon energy [43]. For example, as shown in Table 4, the prepared glasses’ recoded LAC values at 6 MeV are 0.19588, 0.20125, and 0.20195 for T0, TPr, and TPrHo, respectively. As recoded, there is a slight increase in MAC and LAC values using different dopants; sample TPrHo recorded the highest values among the other prepared samples. In comparing the LAC values of the prepared glasses with some of the standard materials, namely RS-253 G18, RS-360, and RS-520, significant shielding performance was observed for the prepared glasses over the standard materials RS-253 G18 and RS-360 (0.069071 and 0.146893 at 6 MeV). On the other hand, the prepared glasses show a slightly lower performance compared with RS-520, this is obviously due to the higher lead oxide content in the RS-520 (70%) compared to 5% lead oxide in sample TPrHo (5%) in Table 4. The recoded MAC and LAC values of the prepared glasses at different energies range between 500 keV and 15 MeV. The good performance of the prepared glasses in terms of good optical, physical, and shielding properties such as good thermal stability, chemical durability, high values of linear and nonlinear refractive index, and shielding effectiveness are all due to the fact that the tellurite-based glass doped with suitable metal oxides and rare earths can form a high-efficiency glass material that can be used in different applications. [2,12,14]. 

**Figure 10:**
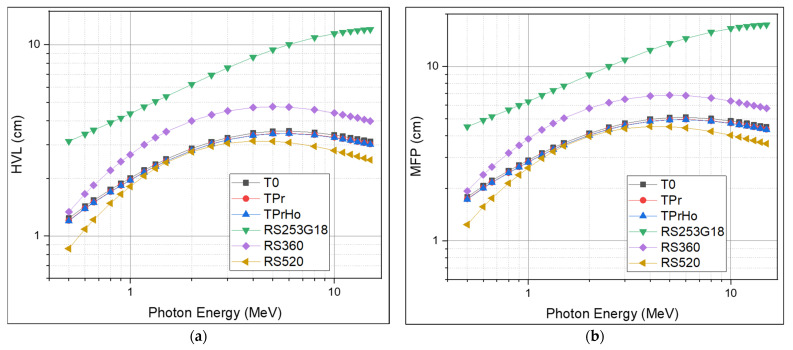
(**a**) Half-value layer of prepared glasses; (**b**) Mean Free Path of prepared glasses.

The HVL represents the absorbance thickness necessary to halve the photon intensity. The mean free path (MFP) is the average distance a photon travels before colliding with a particle in a medium. The HVL and MFP values show how well the gamma radiation is slowed down by the shielding glass material. The HVL and MFP are inversely proportional to the shielding material’s linear attenuation coefficient (LAC); the lower the value, the more effective the material as a shield. The HVL and MFP of the prepared glasses are illustrated in Figure 10a,b. As shown in Figure 10a, there are two fundamental features of the HVL: First, the HVL of all prepared glasses increases with the increasing energy until reaching 6 MeV, it then decreases slowly with the increasing photon energy. For instance, as shown in Table 4, the recorded values at 6 MeV are equal to 3.54, 3.44, 3.43, 10.0, 4.71, and 3.08 cm for T0, TPr, TPrHo, RS254G18, RS360, and RS520, respectively. The recorded increase in HVL is caused by the decrease in the probability of interaction with high-energy photons, which increases the probability of penetrating the samples and necessitates a thicker sample to absorb the same amount of radiation. The second property of the HVL is that it decreases as the absorber density increases; at 6 MeV, the order of the HVL results is as follows: RS520 < TPrHo < TPr < T0 < RS360 < RS254G18. As discussed before, the prepared glasses have LAC values higher than standard materials RS360 and RS254G18, which explain the lower values recorded for the prepared glasses compared with the standard materials. Due to the toxicity of lead oxide, the prepared glasses have a better likelihood of being utilized as an alternative shielding material in medical applications such shielding glass windows and shielding materials used directly on patients undergoing X-ray examinations.

The shielding effectiveness of the prepared glasses can also be investigated in terms of radiation protection efficiency (RPE) [44].

(10) $$\mathrm{RPE}\%=1-e^{-\mu x}$$

**Figure 11:**
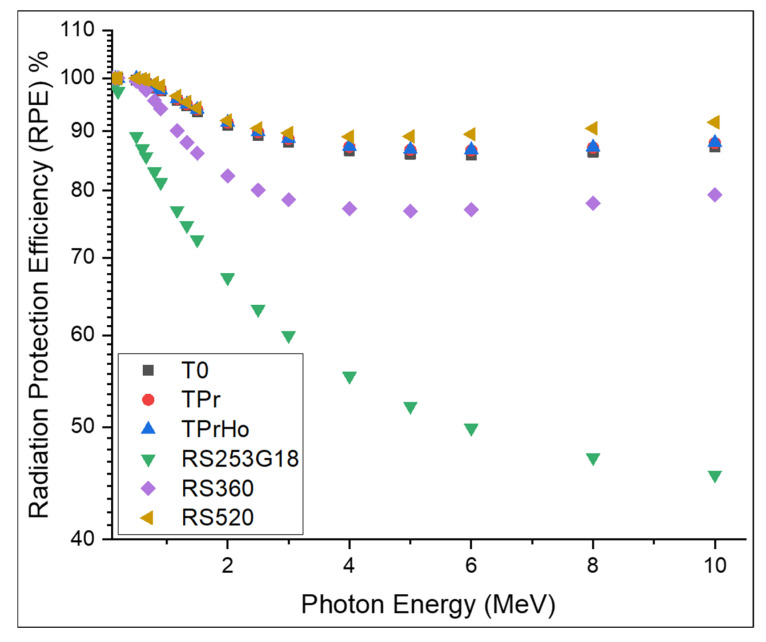
RPE% with photon energy (in MeV) of the prepared glasses.

Figure 11 shows the RPE percentage of the prepared glasses with a thickness of 10 cm at photon energies ranging between 0.1 and 10 MeV. As shown in Figure 11, the RPE decreases with the increasing energy. For instance, the RPE percent decreased from 100 to 87.2% for T0, 100 to 87.9% for TPr, 100 to 88% for TPrHo, from 100 to 45.4% for RS-253G18, from 100 to 79.3% for RS-360, and from 100 to 91.5% for RS-520. As shown in Figure 11, the prepared glasses have good shielding efficiency compared to RS-254G18 and RS-360 and are slightly lower than RS-520. The 10 cm thickness of the prepared glasses has a shielding efficiency above 90% for energies up to 10 MeV.

## 4. Conclusions

The spectroscopic properties of Pr^3+^-doped and Pr^3+^/Ho^3+^-co-doped multicomponent tellurite glass were investigated. Analysis of experimental data shows that there is energy transfer (ET) from Pr^3+^ to Ho^3+^ ions, which increases the emission of the latter ions by a factor of two at 1200 nm. The Pr^3+^−Ho^3+^ down-conversion ET, realized by two successive processes by cross-relaxation (CR), with the Pr^3+^:^1^G_4_ level as an intermediate state, is the cause of the extra emission, which was seen under the 445 nm illumination. This mechanism populates the Ho^3+^:^5^I_6_ level by Pr^3+^(^3^P_0_→^1^G_4_)→Ho^3+^(^5^I_8_→^5^I_6_) and Pr^3+^(^1^G_4_→^3^H_4_)→ Ho^3+^(^5^I_8_→^5^I_6_) pathways, with the excess energies dissipated in the glass matrix. A relatively low ET efficiency (29%) obtained for our Pr^3+/^Ho^3+^ co-doped tellurite glass can be significantly increased by optimizing the concentrations of both ions. The results of luminesces properties of the prepared glasses showed good performance as a laser source for photodynamic therapy (PDT) treatment procedures. In addition to that, the prepared glasses can also be considered as shielding material.

## Figures and Tables

**Table 1:** The sample codes, compositions, molar weight, experimental densities, and the calculated concentrations of lanthanide ions of the investigated samples.

Sample Code	Glass Composition in Mol%	Molar Mass (g/mol)	Density (g/cm^3^)	N_Ln_ (10^20^ cm^−3^)
T0	78TeO_2_-10Nb_2_O_5_-5PbO-5Li_2_O-1La_2_O_3_-1PbF_2_	169.43	5.80	-
TPr	78TeO_2_-10Nb_2_O_5_-5PbO-5Li_2_O-1La_2_O_3_-1PbF_2_3-Pr_2_O_3_	171.85	5.93	N_Pr_ = 6.30
TPrHo	78TeO_2_-10Nb_2_O_5_-5PbO-5Li_2_O-1La_2_O_3_-1PbF_2_-1.5Pr_2_O_3_-1.5Ho_2_O_3_	172.04	5.94	N_Pr_ = 3.16 N_Ho_ = 2.76

**Table 2:** The experimental and calculated oscillator strengths and Judd–Ofelt intensity parameters for Ho^3+^ ions in sample TPrHo.

Transition from ^5^I_8_ to:	Energy (cm^−1^)	Oscillator Strength *f* (10^−6^)	
		*f_exp_*	*f_cal_*
^5^I_6_	8564	0.74	0.95
^5^I_5_	11,178	0.18	0.18
^5^F_5_	15,427	3.02	2.94
^5^S_2_+^5^F_4_	18,501	4.13	3.73
^5^G_6_	22,051	22.76	22.76
^5^G_5_	23,887	3.43	3.56
Ω_2_ = 3.24-, Ω_4_ = 1.64-, Ω_6_ = 1.10 × 10^−20^ cm^2^; *_δrms_* = 0.19 × 10^−6^ Ω_2_ = 6.72-, Ω_4_ = 2.21-, Ω_6_ = 8.74 × 10^−20^ cm^2^ [24] Ω_2_ = 1.24-, Ω_4_ = 7.00-, Ω_6_ = 2.80 × 10^−20^ cm^2^ [28]			

**Table 3:** Lifetimes of Pr^3+^ and Ho^3+^ emission bands obtained experimentally (τ_eff_) as well as the calculated values using the J-O approach (τ_rad_).

Sample Code	Pr^3+^		Ho^3+^ and Pr^3+^		
	^3^P_0_ Lifetime		(^3^F_4_+^5^S_2_) Lifetime		^5^I_6_ Lifetime
	τ_eff_ (μs)	τ_rad_ (μs)	τ_eff_ (μs)	τ_rad_ (μs)	τ_rad_ (μs)
TPr	2.47 [28]	9.43 [28]	-	-	-
TPrHo	2.75	10.01	5.49	301	3.09

**Table 4:** The recoded MAC, LAC, HVL, and MFP values of the prepared glasses at 6 MeV.

Shielding Parameters	T0	TPr	TPrHo	RS253G18	RS-360	RS-520
MAC (cm^2^/g^−1^)	0.03377	0.03394	0.03400	0.02741	0.04080	0.04326
LAC (cm^−1^)	0.19588	0.20125	0.20195	0.06907	0.14689	0.22493
HVL (cm)	3.53793	3.44344	3.43155	10.03314	4.71772	3.08094
MFP (cm)	5.10524	4.96889	4.95173	14.47783	6.80768	4.44580

## Data Availability

Not applicable.
